# A Self‐Designed Endobutton Installation Device for Coracoclavicular Stabilization in Acute Rockwood Type III Acromioclavicular Joint Dislocation

**DOI:** 10.1111/os.13995

**Published:** 2024-01-17

**Authors:** Ma Jie, Tang Yang, Wang Xiang

**Affiliations:** ^1^ Shanghai Key Laboratory of Orthopaedic Implants, Department of Orthopaedic Surgery, Shanghai Ninth People's Hospital Shanghai JiaoTong University School of Medicine Shanghai China

**Keywords:** Acromioclavicular Joint, Coracoclavicular Ligament, Dislocation, Endobutton, Stabilization

## Abstract

**Objective:**

Endobutton technique could provide flexible coracoclavicular (CC) stabilization for acromioclavicular joint (ACJ) dislocation and achieved good clinical outcomes. However, the difficult part of this technique was placement of the Endobutton to the coracoid base. In this study, we designed an Endobutton installation device to place the Endobutton at the coracoid base. And we examined the clinical and radiographic outcomes of patients with acute Rockwood type III ACJ dislocation repaired with Endobutton using this device.

**Methods:**

We designed an Endobutton installation device to place the Endobutton at the coracoid base to achieve CC stabilization. We retrospectively reviewed 42 patients with acute Rockwood type III ACJ dislocation who underwent CC stabilization with Endobuttons placed either using this novel device (group I, n = 19) or the traditional technique (CC stabilization without using special device, group II, n = 23) from January 2015 to April 2020. The two groups were compared regarding the operative time, intraoperative blood loss, and clinical and radiologic outcomes at final follow‐up. The operation‐related complications were also evaluated. The Student's t test and the Mann–Whitney U‐test were used to compare differences in continuous variables. Differences in categorical variables were assessed with either the Pearson's chi‐squared test or Fisher's exact test.

**Results:**

Forty‐two patients were clinically followed up for a minimum of 12 months. Compared with group II, group I had a significantly shorter mean operative time (56.05 ± 7.82 min vs. 65.87 ± 7.43 min, *p* < 0.01) and significantly lesser mean intraoperative blood loss (67.89 ± 14.75 mL vs. 94.78 ± 25.01 mL, *p* < 0.01). At final follow‐up, there were no significant differences between the two groups in the visual analog scale score for pain, Oxford Shoulder Score, Disabilities of the Arm, Shoulder, and Hand score, and postoperative CC distance of the affected side. Loss of reduction occurred in four patients in group I and three patients in group II (*p* = 0.68); there were no other operation‐related complications in either group.

**Conclusions:**

The Endobutton installation device makes placement of the Endobutton at the coracoid base easier and achieves satisfactory clinical and radiologic outcomes without additional complications in acute Rockwood type III ACJ dislocation.

## Graphical abstract

There have been no studies of special devices used for placement of Endobutton for the CC stabilization. We presented a self‐designed Endobutton installation device, which can easily and efficiently place the Endobutton to the coracoid base for the CC stabilization in the acute Rockwood type III ACJ dislocation.

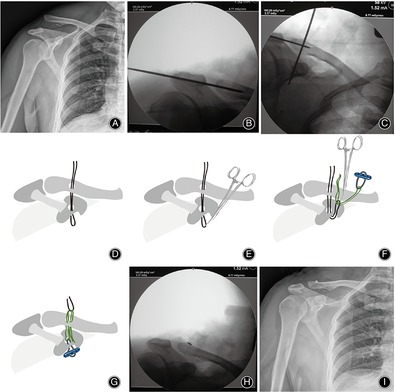

## Introduction

Acromioclavicular joint (ACJ) dislocation is a common injury among the active population.^1^, ^2^ In acute Rockwood type III ACJ dislocation, the coracoclavicular (CC) ligament is completely torn; this disruption of the CC ligament leads to vertical instability because of the downward pull of the weight of the arm and the superior pull of the trapezius muscle.^3^, ^4^ As a result, nonsurgical treatment of acute Rockwood type III ACJ dislocation may result in high rates of pain and shoulder dysfunction.^5^, ^6^ Therefore, surgery is recommended for acute Rockwood type III ACJ dislocation, especially in young active patients.^7^

The surgical techniques used to treat acute Rockwood type III ACJ dislocation include temporary K‐wire transfixation, CC screw fixation, hook plate fixation, and Endobutton placement.^8^, ^9^, ^10^ However, each surgical technique has advantages and disadvantages, and there is no consensus regarding the “gold standard” of fixation for acute Rockwood type III ACJ dislocation.^11^ CC ligament reconstruction is currently recommended for ACJ dislocation because the CC ligament plays a crucial role in the physiological function of the ACJ.^12^, ^13^ CC stabilization using Endobuttons placed either *via* open surgery or arthroscopically reportedly achieves good clinical and radiological outcomes.^14^, ^15^ However, the difficult part of the Endobutton technique for CC stabilization was placement of the Endobutton at the coracoid base,^16^ which often required repeated attempts. Therefore, more soft tissue stripping, the operative time and intraoperative blood loss were needed to finish the CC stabilization.

Herein, we present a self‐designed Endobutton installation device designed to place the Endobutton at the coracoid base. The purpose of the present study is to examine the clinical and radiographic outcomes of patients with acute Rockwood type III ACJ dislocation repaired with Endobutton using this device.

## Materials and Methods

### Self‐Designed Endobutton Installation Device

**FIGURE 1:**
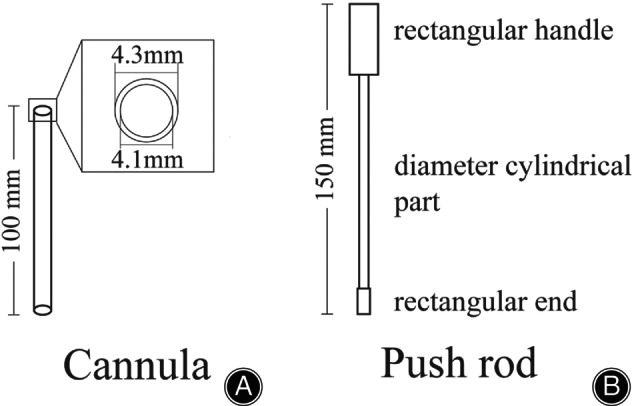
Illustration of the self‐designed Endobutton installation device. (A) The cannula is 100 mm long and its outer and inner diameters are 4.3 mm and 4.1 mm, respectively. (B) The pushing rod is 150 mm long and is composed of a 30 mm long, 10 mm wide, and 2.0 mm thick rectangular handle; a 110 mm long cylindrical part with a 2.0 mm diameter; and a 10 mm long, 2.5 mm wide, and 1.5 mm thick rectangular end.

**FIGURE 2:**
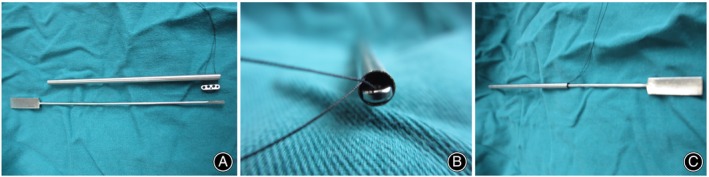
The self‐designed Endobutton installation device used to place an Endobutton at the coracoid base. (A) Lateral view of the cannula, Endobutton, and pushing rod. (B) Transverse view of the cannula and Endobutton. (C) Lateral view of the Endobutton being passed through the cannula using the pushing rod.

The difficult part of Endobutton technique for CC stabilization was placement of the Endobutton to the coracoid base. Therefore, we designed an Endobutton installation device to easily place the Endobutton at the coracoid base for CC stabilization. The self‐designed Endobutton installation device is composed of a cannula and a pushing rod (Patent number: ZL201821603597.9). The cannula is 100 mm long and has outer and inner diameters of 4.3 mm and 4.1 mm, respectively, through which the Endobutton (12 mm long, 3.75 mm wide, 1.5 mm thick; Johnson & Johnson, Piscataway, NJ, USA) can pass. The pushing rod is 150 mm long and has a rectangular handle (30 mm long, 10 mm wide, 2.0 mm thick), cylindrical part (110 mm long, 2.0 mm diameter), and rectangular end (10 mm long, 2.5 mm wide, 1.5 mm thick) (Figure 1). The Endobutton installation device is made of medical stainless steel and was produced by Double Medical Technology Inc., Xiamen, China (Figure 2).

### Study Population

This retrospective study included patients with acute Rockwood type III ACJ dislocation who received CC stabilization using either the Endobutton installation device (group I) or the traditional Endobutton placement technique (group II) from January 2015 to April 2020. The inclusion criteria were (1): acute Rockwood type III ACJ dislocation that occurred within 2 weeks of surgery; (2) consent for surgical treatment; (3) postoperative follow‐up of at least 12 months. The exclusion criteria were: (1) age younger than 18 years; (2) history of surgery on the affected shoulder; (3) concomitant fracture around the affected shoulder; (4) chronic ACJ dislocation; (5) severe osteoporosis. Surgery was recommended for patients with high activity levels. Forty‐two patients with acute Rockwood type III ACJ dislocation consented to surgical treatment and were included in this study. All surgeries were performed by two surgeons (J.M. and X.W.) at a single institution. This study was approved by the Ethics Committee of the hospital (approval number: SH9H‐2023‐T89‐1).

### Surgical Technique

After the induction of general anesthesia, the patients were placed in the beach‐chair position. A 5‐cm transverse incision was made over the lateral third of the clavicle extending toward the ACJ. The ACJ reduction was achieved and maintained by temporary K‐wire fixation across the ACJ. The anterior deltoid muscles were bluntly dissected along the deltoid fibers from the clavicle to the tip of the coracoid. The soft tissue under the coracoid base was then pushed away with the surgeon's fingers. A director was placed from the middle of the clavicle (20–30 mm from the distal end of the clavicle) to the middle of the coracoid base under X‐ray guidance.

**FIGURE 3:**
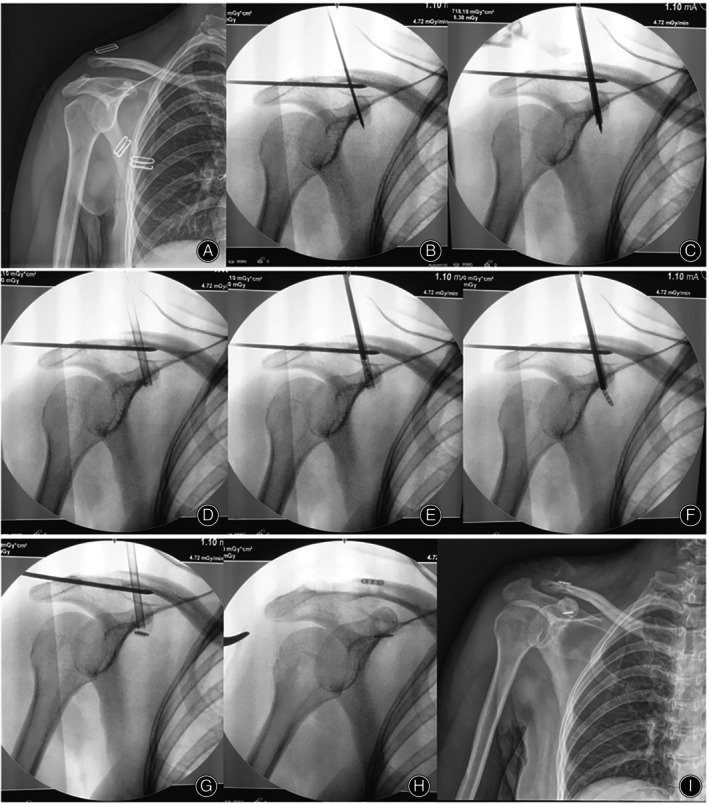
A 42‐year‐old woman with Rockwood type III ACJ dislocation. (A) Preoperative radiographs. (B) The ACJ is reduced and maintained by temporary K‐wires and a director is placed from the middle of the clavicle to the middle of the coracoid base. (C) A 4.6‐mm bony tunnel is drilled through the director. (D) The cannula is inserted through the bony tunnel from the clavicle to the coracoid. (E) A suture was placed through the Endobutton loop, and the Endobutton was then put into the cannula. (F) The Endobutton was placed at the coracoid base by the pushing rod. (G) The Endobutton gradually closed to the coracoid base by pulling the suture. (H) The second Endobutton plate without loops is placed in the loop above the clavicle. (I) Postoperative radiograph obtained to assess the reduction.

In group I, a 4.6‐mm bony tunnel was drilled through the director. The cannula was inserted into the bony tunnel from the clavicle surface to the coracoid base. A suture was placed through the Endobutton loop, and the Endobutton was then put into the cannula. Under X‐ray guidance, the Endobutton was placed at the coracoid base by the pushing rod. The Endobutton loop was brought to the clavicle surface by pulling the suture. Another Endobutton was inserted through the Endobutton loop to reconstruct the CC ligament (Figure 3).

**FIGURE 4:**
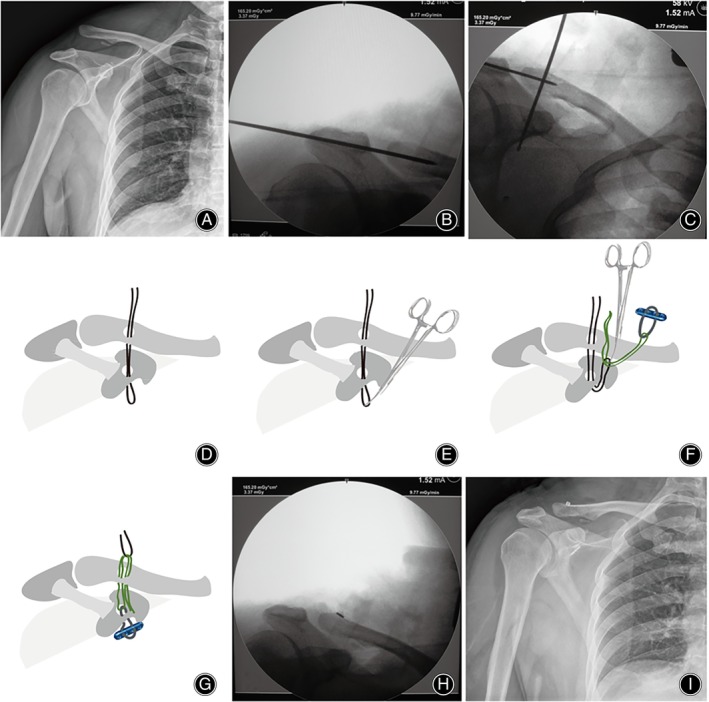
A 46‐year‐old man with Rockwood type III ACJ dislocation. (A) Preoperative radiographs. (B) The ACJ reduction is achieved and maintained by the placement of temporary K‐wires across the ACJ. (C) A director is placed from the middle of the clavicle to the middle of the coracoid base. (D) Illustration showing the stainless steel suture folded into double strands. A 4.2‐mm bony tunnel is drilled through the director and the middle fold of the steel suture is passed through the bony tunnel to the coracoid base. (E) Illustration of the middle fold of the steel suture being taken to the incision site by the vascular forceps. (F) Illustration showing the No. 1 Ethibond suture passing through the loop of the Endobutton and through the middle fold of the steel suture. (G) Illustration of the Endobutton placed at the coracoid base and the loop brought to the surface of clavicle by pulling the middle fold of the steel suture and the Ethibond suture. (H) The second Endobutton plate without loops is placed in the loop above the clavicle. (I) Postoperative radiograph obtained to assess the reduction.

In group II, a 4.2‐mm bony tunnel was drilled through the director. A stainless steel suture (M649G, Ethicon Inc., Somerville, NJ, USA) was folded into double strands. The middle fold of the steel suture was passed through the bony tunnel to the coracoid base and was taken to the incision site by vascular forceps. No. 1 Ethibond suture (Ethicon Inc.) was then passed through the loop of the Endobutton and through the middle fold of the steel suture. The Endobutton was placed at the coracoid base and the loops were brought to the surface of clavicle by pulling the middle fold of the steel suture and the Ethibond suture. Another Endobutton was inserted through the Endobutton loop to reconstruct the CC ligament (Figure 4).

### Postoperative Rehabilitation

Patients were encouraged to begin passive movement of the shoulder, including pendulum exercises, self‐assisted circumduction exercises, and gradual passive range of motion (ROM) exercises on the first day postoperatively. Active ROM of the shoulder was encouraged at 1 week postoperatively when the pain was sufficiently relieved. A shoulder sling was used for 4 weeks postoperatively. Patients were instructed to avoid lifting, carrying, pushing, and pulling with strong force for 8 weeks postoperatively.

### Clinical and Radiographic Evaluation

The operative time (minutes) was recorded as the time from skin incision to the closure of the wound. Intraoperative blood loss (ml) was also recorded. The patients were followed up at a minimum of 12 months postoperatively to evaluate the clinical and radiologic outcomes. The clinical outcomes were evaluated using the visual analog scale (VAS) score for pain, Oxford Shoulder Score, and Disabilities of the Arm, Shoulder, and Hand (DASH) score. For the evaluation of radiologic outcomes, the CC distances (CCD) of the affected and contralateral sides were measured on anteroposterior radiographs as described previously.^17^ Briefly, the CCD was measured between the uppermost border of the coracoid process and the opposing clavicular surface. CCD measurements were performed by two clinicians (J.M. and Y.T.). Operation‐related complications such as incision infection, loss of reduction, re‐dislocation, implant loosening, pleural injury, neurovascular injury, and iatrogenic fracture were recorded. Loss of reduction and re‐dislocation were defined as increases in the CCD with respect to the contralateral side of 50%–100% and >100%, respectively, at final follow‐up.^18^, ^19^

### Statistical Analysis

SPSS software (version 23.0; IBM Corp., Armonk, NY, USA) was used for statistical evaluations. The Student's t test and the Mann–Whitney U‐test were used to compare differences in continuous variables. Differences in categorical variables were assessed with either the Pearson's chi‐squared test or Fisher's exact test. The level of significance was *p* < 0.05 for all tests.

## Results

### Patient Demographics

The patient demographics were detailed in Table 1. There were no significant differences between the two groups in terms of age, sex, affected side, injury type, duration from injury to operation, and duration of follow‐up (all *p* > 0.05).

**TABLE 1:** Patient demographics.

Demographics	Group I (n = 19)	Group II (n = 23)	*p*‐value
Gender			
Male	13	16	1.00
Female	6	7	
Age (years, mean ± SD)	43.26 ± 8.41	40.26 ± 10.02	0.16
Side of injury			
Right	10	10	0.76
Left	9	13	
Injury type			
Vehicle accident	7	10	0.88
Falls from a height	8	8	
Direct violent injury	4	5	
Duration to operation (days, mean ± SD)	3.74 ± 1.91	3.65 ± 1.36	0.80
Follow‐up time (months, mean ± SD)	18.68 ± 3.77	17.13 ± 4.59	0.15

Abbreviation: SD, standard deviation.

Of the 42 patients with acute Rockwood type III ACJ dislocation, 19 patients (13 men and six women) were treated using the Endobutton installation device to place the Endobutton at the coracoid base for CC stabilization (group I). In group I, the mean age was 43.26 years (range 28–56 years); the dislocation occurred on the right side in 10 patients and the left side in nine; seven, eight, and four injuries were caused by motor vehicle accidents, falls from a height, and direct traumatic injuries, respectively; the mean time from injury to surgery was 3.74 days (range 2–10 days); and the mean follow‐up was 16.68 months (range 13–24 months).

Twenty‐three patients (16 men and seven women) with acute Rockwood type III ACJ dislocation underwent CC stabilization using the traditional Endobutton placement technique (group II). In group II, the mean age was 40.26 years (range 22–61 years); the dislocation occurred on the right side in 10 patients and the left side in 13; 10, eight, and five injuries were caused by motor vehicle accidents, falls from a height, and direct traumatic injuries, respectively; the mean time from injury to surgery was 3.65 days (range 2–7 days); and the mean follow‐up was 17.13 months (range 12–26 months).

### Clinical and Radiographic Outcomes

The operative details were summarized in Table 2. The mean operative time was 56.05 ± 7.82 minutes (range 43–70 minutes) in group I and 65.87 ± 7.43 minutes (range 54–87 minutes) in group II (*p* < 0.01). The intraoperative blood loss was 67.89 ± 14.75 ml (range 50–100 ml) in group I and 94.78 ± 25.01 ml (range 50–150 ml) in group II (*p* < 0.01).

**TABLE 2:** Operation‐related factors.

	Group I (n = 19)	Group II (n = 23)	*p*‐value
Operative time (min, mean ± SD)	56.05 ± 7.82	65.87 ± 7.43	<0.01
Blood loss (mL, mean ± SD)	67.89 ± 14.75	94.78 ± 25.01	<0.01

Abbreviation: SD, standard deviation.

The clinical outcomes were shown in Table 3. At a minimum of 12 months postoperatively, all 42 patients had satisfactory clinical outcomes. At final follow‐up in groups I and II, the average VAS scores for pain were 1.1 ± 0.9 (range, 0–3) and 1.3 ± 0.9 (range, 0–3), respectively (p = 0.57), the mean Oxford Shoulder Scores were 15.89 ± 3.41 (range, 12–24) and 15.78 ± 3.58 (range, 12–26), respectively, (p = 0.82), and the mean DASH scores were 2.11 ± 1.01 (range, 0–4.16) and 2.28 ± 1.02 (range, 0–3.83), respectively, (*p* = 0.63).

**TABLE 3:** Clinical outcomes.

	Group I (n = 19)	Group II (n = 23)	*p*‐value
VAS score (mean ± SD)	1.11 ± 0.88	1.26 ± 0.86	0.57
Oxford shoulder scores (mean ± SD)	15.89 ± 3.41	15.78 ± 3.58	0.82
DASH score (mean ± SD)	2.11 ± 1.01	2.28 ± 1.02	0.63

Abbreviations: DASH score, disabilities of the arm, shoulder and hand score; SD, standard deviation; VAS, visual analog scale.

The radiologic outcomes were shown in Table 4. All patients achieved satisfactory radiologic outcomes at final follow‐up. There were significant differences between the pre‐ and postoperative CCD on the injured side in both groups I and II (*p* < 0.01). The mean postoperative CCD of the injured side did not significantly differ between groups I and II (*p* = 0.30).

**TABLE 4:** Radiographic outcomes.

	Pre‐op		Post‐op		*p*‐value		
	group I (n = 19)	group II (n = 23)	group I (n = 19)	group II (n = 23)			
CCD (mean ± SD, mm)	17.36 ± 1.61	17.57 ± 1.73	9.59 ± 2.40	9.46 ± 1.87	P1 < 0.01	P2 < 0.01	P3 = 0.30

Abbreviations: CCD, coracoclavicular distance; P1, pre–post CCD of group I; P2, pre–post CCD of group II; P3, post CCD of group I‐group II; SD, standard deviation.

### Postoperative Complications

At final follow‐up, a loss of reduction was radiographically confirmed in four patients in group I and three patients in group II (*p* = 0.68). In group I, the mean preoperative CCD of the affected side was 17.55 ± 0.91 mm (range, 17.1–18.5 mm), the mean postoperative CCD of the affected side was 13.85 ± 0.89 mm (range, 12.5–14.6 mm), and the mean CCD of the contralateral side was 8.55 ± 0.55 mm (range, 8–9 mm). In group II, the mean preoperative CCD of the affected side was 17.87 ± 0.90 mm (range, 17.2–18.9 mm), the mean postoperative CCD of the affected side was 13.87 ± 0.81 mm (range, 13.3–14.8 mm), and the mean CCD of the contralateral side was 8.4 ± 0.20 mm (range, 8.2–8.6 mm). The VAS score for pain in the injured shoulder was 2–3 (indicating mild pain) in seven patients, but no patients required additional analgesic medication. There was no radiological evidence of re‐dislocation or implant loosening at final follow‐up in either of the two groups. No patients had incision infection, pleural injury, neurovascular injury, or iatrogenic fracture.

## Discussion

### Technique Advantages

To our knowledge, this is the first report of a self‐designed Endobutton installation device for placing the Endobutton at the coracoid base to achieve CC stabilization in acute Rockwood type III ACJ dislocation. In our study, this technique enabled us to easily and effectively place the Endobutton at the coracoid base and achieve satisfactory clinical and radiologic outcomes without severe complications.

### Technical Characteristic

The following three surgical techniques are frequently used for CC stabilization in clinical practice^8^, ^20^, ^21^, ^22^: (1) ACJ fixation (static techniques: K‐wires [no longer used because of complications]; dynamic techniques: hook plate); (2) CC fixation (static techniques: CC screws; dynamic techniques: Endobuttons); and (3) CC ligament reconstruction. However, each of these surgical techniques has complications and the best treatment strategy for CC stabilization remains controversial. The most commonly used surgical procedure is internal fixation with the Endobutton technique,^23^, ^24^ which can be performed either *via* open surgery, mini‐open surgery, or arthroscopically.^15^, ^16^, ^21^ Endobuttons technique provide flexible CC stabilization for ACJ dislocation.^25^, ^26^ However, as described above in the Surgical Technique section (Figure 3), the difficult part of the traditional Endobutton technique for CC stabilization is placement of the Endobutton at the coracoid base,^16^ which often requires repeated attempts. The use of the traditional Endobutton technique may be associated with problems such as more soft tissue stripping, operative time, and intraoperative blood loss. However, to our knowledge, no studies have reported the use of special devices for Endobutton placement. We designed an Endobutton installation device to place the Endobutton at the coracoid base for CC stabilization. During the operation, instead of 4.2‐mm bony tunnel in the traditional Endobutton technique, a 4.6‐mm bony tunnel from the clavicle surface to the coracoid base was needed for the annula pass through. And then, the Endobutton was easily placed at the coracoid base through the annula.

### Application of the Technique

In our study, we retrospectively analyzed 19 and 23 patients with acute Rockwood type III ACJ dislocation treated with Endobutton placement using the Endobutton installation device (group I) and the traditional technique (group II), respectively. During the operation, a mini‐open technique was used in both groups, with a finger used to protect the neurovascular structures under the coracoid base when the director was placed and the bony tunnel was drilled. This procedure seemed to be safe and simple compared with arthroscopy or surgery under extensive radiographic guidance. Additionally, our results suggest that the Endobutton installation device easily and effectively placed the Endobutton at the coracoid base. And, the operative time and intraoperative blood loss were significantly reduced in group I compared with group II. At final follow‐up, all 42 patients achieved satisfactory clinical outcomes, with no significant differences between groups I and II in the mean VAS score for pain, mean Oxford Shoulder Score, and mean DASH score. Moreover, on radiographs obtained at final follow‐up, the postoperative CCD was significantly decreased compared with the preoperative CCD of the injured side in both groups, and the postoperative CCD of the injured side was similar in the two groups. There was a similar incidence of loss of reduction in both groups. In both groups, we only reconstructed the conical ligaments, which lacks horizontal stability.^27^ Therefore, the loss of reduction might have occurred mainly because the reconstruction of the conical ligaments was not able to completely stabilize the ACJ.^18^ However, as previously reported, this loss of reduction was not significantly associated with clinical outcomes.^26^, ^28^ The patients with loss of reduction still achieved satisfactory clinical outcomes, and no further complications occurred in either group in the present study.

### Technique Risks and Strategies

Despite the good outcomes achieved in the present study, the Endobutton installation device has some disadvantages. Firstly, the bony tunnels are drilled using a 4.6‐mm drill instead of a 4.2‐mm drill. Although there were no clavicle and coracoid fractures in our study, the use of a drill with a larger diameter carries a risk of this kind of complication. We think that these complications were prevented because the bony tunnels were drilled through the middle of the distal clavicle and the coracoid base, which are wide enough to drill 4.5–5.0‐mm bony tunnels.^29^ Secondly, there is a theoretical risk that an Endobutton is more likely to move into a 4.6‐mm bony tunnel than a 4.2‐mm bony tunnel. However, no such complications occurred in our study. We consider that the incidence of implant loosening may have been reduced by implementation of the following two procedures.^1^ The Endobutton loop was kept under appropriate tension. With the aid of direct vision and radiographic monitoring, we confirmed that the ACJ dislocation was completely reduced and maintained by temporary K‐wires placed across the ACJ. The bony tunnel was then drilled and the length was carefully measured. Based on this measurement, we chose a loop that was the same size or one size larger than the tunnel length. After the loop was brought to the clavicle surface, another Endobutton was inserted through the loop.^2^ The Endobutton was placed horizontally above or below the center of the tunnels in the distal clavicle and the coracoid base. Thirdly, the drilling of a bony tunnel through the coracoid base carries the risk of pleural injury and neurovascular injury. To prevent such injuries, the surgeons used their fingers to push away the soft tissue under the coracoid base. The surgeons then placed a finger at the coracoid base when the director was placed and the bony tunnels were drilled. These procedures may avoid pleural and neurovascular injuries.

## Limitations

Our study has several limitations. This study was a single‐center retrospective study with a low level of evidence and a small number of patients. Thus, the present results require confirmation in a multicenter study with a large sample size. Additionally, there may have been biases related to coding that may have influenced the identification of potentially eligible patients. To control for the bias related to coding, we carefully code the ACJ dislocation based on the Rockwood classification in the future study.

### Prospects for Clinical Application

The endobutton installation device may be a good option for placing the Endobutton at the coracoid base to achieve CC stabilization, such as in the ACJ dislocation and Neer type IIB lateral clavicle fractures. It makes placement of the Endobutton at the coracoid base easier with less soft tissue stripping, the operative time and intraoperative blood loss. However, a drill with a larger diameter carries risk of clavicle and coracoid fractures in this surgical procedure. To avoid the intraoperative clavicle and coracoid fractures, the surgeon needed to manipulate carefully to maintain the bony tunnels were drilled through the middle of the distal clavicle and the coracoid base.

## Conclusion

In conclusion, the Endobutton installation device makes placement of the Endobutton at the coracoid base easier and achieves satisfactory clinical and radiologic outcomes without additional complications in acute Rockwood type III ACJ dislocation.

## Author's Contribution

JM were major contributors in writing the manuscript and performed the surgery. YT suggested the idea and measured/analyzed the data. XW suggested the idea and performed the surgery. All authors read and approved the final manuscript.

## Conflict of Interest Statement

The authors declare that they have no competing interests.

## References

[1] Haugaard KB , Bak K , Seem K , Holmich P , Barfod KW . Rockwood type III is the most common type of acromioclavicular joint dislocation: a prospective cohort study investigating the incidence and epidemiology of acute acromioclavicular joint dislocations in an urban population. Shoulder Elbow. 2023;15(5):505–512. 37811384 10.1177/17585732221123314 PMC10557934

[2] Skjaker SA , Enger M , Engebretsen L , Brox JI , Boe B . Young men in sports are at highest risk of acromioclavicular joint injuries: a prospective cohort study. Knee Surg Sports Traumatol Arthrosc. 2021;29(7):2039–2045. 32270265 10.1007/s00167-020-05958-x PMC8225525

[3] Cook JB , Shaha JS , Rowles DJ , Bottoni CR , Shaha SH , Tokish JM . Clavicular bone tunnel malposition leads to early failures in coracoclavicular ligament reconstructions. Am J Sports Med. 2013;41(1):142–148. 23139253 10.1177/0363546512465591

[4] Ferreira JV , Chowaniec D , Obopilwe E , Nowak MD , Arciero RA , Mazzocca AD . Biomechanical evaluation of effect of coracoid tunnel placement on load to failure of fixation during repair of acromioclavicular joint dislocations. Arthroscopy. 2012;28(9):1230–1236. 22560485 10.1016/j.arthro.2012.02.004

[5] Giai Via R , Bosco F , Giustra F , Lavia AD , Artiaco S , Risitano S , et al. Acute Rockwood type III ACJ dislocation: conservative vs surgical approach. A systematic review and meta‐analysis of current concepts in literature. Injury. 2022;53(10):3094–3101. 35945090 10.1016/j.injury.2022.07.050

[6] De Rooij PP , Van Lieshout EMM , Schurink IJ , Verhofstad MHJ , group ACJis . Current practice in the management of acromioclavicular joint dislocations; a national survey in The Netherlands. Eur J Trauma Emerg Surg. 2021;47(5):1417–1427. 32535639 10.1007/s00068-020-01414-0 PMC8476372

[7] Korsten K , Gunning AC , Leenen LP . Operative or conservative treatment in patients with Rockwood type III acromioclavicular dislocation: a systematic review and update of current literature. Int Orthop. 2014;38(4):831–838. 24178060 10.1007/s00264-013-2143-7 PMC3971277

[8] Beris A , Lykissas M , Kostas‐Agnantis I , Vekris M , Mitsionis G , Korompilias A . Management of acute acromioclavicular joint dislocation with a double‐button fixation system. Injury. 2013;44(3):288–292. 23352675 10.1016/j.injury.2013.01.002

[9] Yoo YS , Khil EK , Im W , Jeong JY . Comparison of hook plate fixation versus arthroscopic coracoclavicular fixation using multiple soft anchor knots for the treatment of acute high‐grade acromioclavicular joint dislocations. Arthroscopy. 2021;37(5):1414–1423. 33340675 10.1016/j.arthro.2020.12.189

[10] Nolte PC , Lacheta L , Dekker TJ , Elrick BP , Millett PJ . Optimal Management of Acromioclavicular Dislocation: current perspectives. Orthop Res Rev. 2020;12:27–44. 32184680 10.2147/ORR.S218991 PMC7062404

[11] Hachem AI , Rondanelli R , Costa G , Verdalet I , Ezzeddine H , Rius X . Arthroscopically assisted comprehensive double cerclage suture fixation technique for acute acromioclavicular joint separation. Arthrosc Tech. 2020;9(10):e1495–e1504. 33134051 10.1016/j.eats.2020.06.012 PMC7587229

[12] Stucken C , Cohen SB . Management of acromioclavicular joint injuries. Orthop Clin North Am. 2015;46(1):57–66. 25435035 10.1016/j.ocl.2014.09.003

[13] Filho RB , Freitas MM , Nunes RHR , Tenor Junior AC , Costa MPD , Roberto RA . Acromioclavicular, coracoclavicular and medial Coracoclavicular ligaments assessment in acromioclavicular dislocation. Rev Bras Ortop. 2021;56(6):777–783. 10.1055/s-0040-1719088 PMC8651455 34900107

[14] Torkaman A , Bagherifard A , Mokhatri T , Haghighi MH , Monshizadeh S , Taraz H , et al. Double‐button fixation system for Management of Acute Acromioclavicular Joint Dislocation. Arch Bone Jt Surg. 2016;4(1):41–46. 26894217 PMC4733234

[15] Pan Z , Zhang H , Sun C , Qu L , Cui Y . Arthroscopy‐assisted reconstruction of coracoclavicular ligament by Endobutton fixation for treatment of acromioclavicular joint dislocation. Arch Orthop Trauma Surg. 2015;135(1):9–16. 25421528 10.1007/s00402-014-2117-2 PMC4281352

[16] Hu F , Han S , Liu F , Wang Z , Jia H , Wang F , et al. A modified single‐endobutton technique combined with nice knot for treatment of Rockwood type III or V acromioclavicular joint dislocation. BMC Musculoskelet Disord. 2022;23(1):15. 34980065 10.1186/s12891-021-04915-0 PMC8725473

[17] Mori D , Yamashita F , Kizaki K , Funakoshi N , Mizuno Y , Kobayashi M . Anatomic coracoclavicular ligament reconstruction for the treatment of acute acromioclavicular joint dislocation: minimum 10‐year follow‐up. JBJS Open Access. 2017;2(3):e0007. 10.2106/JBJS.OA.16.00007 PMC6133097 30229219

[18] Yoon JP , Lee BJ , Nam SJ , Chung SW , Jeong WJ , Min WK , et al. Comparison of results between hook plate fixation and ligament reconstruction for acute unstable acromioclavicular joint dislocation. Clin Orthop Surg. 2015;7(1):97–103. 25729525 10.4055/cios.2015.7.1.97 PMC4329540

[19] Zhu Y , Hsueh P , Zeng B , Chai Y , Zhang C , Chen Y , et al. A prospective study of coracoclavicular ligament reconstruction with autogenous peroneus longus tendon for acromioclavicular joint dislocations. J Shoulder Elbow Surg. 2018;27(6):e178–e188. 29397294 10.1016/j.jse.2017.12.009

[20] Baunach D , Eid K , Ricks M , Borbas P . Long‐term clinical and radiological results after hook plate osteosynthesis of lateral clavicle fractures. J Orthop Trauma. 2021;35(7):378–383. 33177428 10.1097/BOT.0000000000002007

[21] Manohara R , Reid JT . Percutaneous endobutton fixation of acute acromioclavicular joint injuries and lateral clavicle fractures. J Clin Orthop Trauma. 2019;10(3):492–496. 31061575 10.1016/j.jcot.2018.10.013 PMC6494760

[22] Yagnik GP , Porter DA , Jordan CJ . Distal clavicle fracture repair using cortical button fixation with coracoclavicular ligament reconstruction. Arthrosc Tech. 2018;7(4):e411–e415. 29942734 10.1016/j.eats.2017.10.012 PMC6011383

[23] Civan O , Atmaca H , Ugur L . Biomechanical comparison of double versus triple button reconstruction techniques in patients with acromioclavicular joint dislocation. Int J Med Robot. 2020;16(1):e2057. 31713270 10.1002/rcs.2057

[24] Wang YC , Yong M , Wei‐zhong Y , Wang H . Surgical treatment of acute Rockwood III acromioclavicular dislocations‐comparative study between two flip‐button techniques. Sci Rep. 2020;10(1):4447. 32157165 10.1038/s41598-020-61488-z PMC7064491

[25] Spoliti M , De Cupis M , Via AG , Oliva F . All arthroscopic stabilization of acute acromioclavicular joint dislocation with fiberwire and endobutton system. Muscles, Ligaments Tendons J. 2014;4(4):398–403. 25767774 PMC4327346

[26] Struhl S , Wolfson TS . Continuous loop double Endobutton reconstruction for acromioclavicular joint dislocation. Am J Sports Med. 2015;43(10):2437–2444. 26260466 10.1177/0363546515596409

[27] Wellmann M , da Silva G , Lichtenberg S , Magosch P , Habermeyer P . Instability pattern of acromioclavicular joint dislocations type Rockwood III: relevance of horizontal instability. Orthopade. 2013;42(4):271–277. 23512005 10.1007/s00132-013-2085-1

[28] Salzmann GM , Walz L , Buchmann S , Glabgly P , Venjakob A , Imhoff AB . Arthroscopically assisted 2‐bundle anatomical reduction of acute acromioclavicular joint separations. Am J Sports Med. 2010;38(6):1179–1187. 20442326 10.1177/0363546509355645

[29] Grantham C , Heckmann N , Wang L , Tibone JE , Struhl S , Lee TQ . A biomechanical assessment of a novel double endobutton technique versus a coracoid cerclage sling for acromioclavicular and coracoclavicular injuries. Knee Surg Sports Traumatol Arthrosc. 2016;24(6):1918–1924. 25073944 10.1007/s00167-014-3198-8

